# Optimization of enzymatic hydrolysis by alcalase and flavourzyme to enhance the antioxidant properties of jasmine rice bran protein hydrolysate

**DOI:** 10.1038/s41598-022-16821-z

**Published:** 2022-07-22

**Authors:** Kanrawee Hunsakul, Thunnop Laokuldilok, Vinyoo Sakdatorn, Wannaporn Klangpetch, Charles S. Brennan, Niramon Utama-ang

**Affiliations:** 1 Division of Product Development Technology, Faculty of Agro-Industry, Chiang Mai University, Chiang Mai, 50100 Thailand; 2 Division of Marine Product Technology, Faculty of Agro-Industry, Chiang Mai University, Chiang Mai, 50100 Thailand; 3 Cluster of High Value Product of Thai Rice and Plant for Health, Chiang Mai University, Chiang Mai, 50100 Thailand; 4 Cluster of Innovative Food and Agro-Industry, Faculty of Agro-Industry, Chiang Mai University, Chiang Mai, 50100 Thailand; 5 Food Innovation and Packaging Center, Chiang Mai University, Chiang Mai, 50100 Thailand; 6 Division of Food Science and Technology, Faculty of Agro-Industry, Chiang Mai University, Chiang Mai, 50100 Thailand; 7 School of Science, RMIT University, Melbourne, VIC 3001 Australia

**Keywords:** Enzymes, Peptides, Proteins

## Abstract

This study aimed to optimize the hydrolysis conditions for producing jasmine rice bran protein hydrolysate (JBH) using response surface methodology (RSM). The independent variables were the ratio of flavourzyme to alcalase (Fl:Al; 0: 100 to 15: 85; 2.84% enzyme concentration) and hydrolysis time (60–540 min). The optimum hydrolysate was obtained at an Fl:Al ratio of 9.81: 90.19 for 60 min, since it enabled high amounts of protein, high antioxidant activity and more low molecular weight proteins. The experimental values obtained were a degree of hydrolysis (DH) of 7.18%, a protein content of 41.73%, an IC_50_ for DPPH of 6.59 mg/mL, an IC_50_ for ABTS of 0.99 mg/mL, FRAP of 724.81 mmol FeSO_4_/100 g, and 322.35 and 479.05 mAU*s for peptides with a molecular weight of < 3 and 3–5 kDa, respectively. Using a mixture of enzymes revealed the potential of mixed enzymes to produce JBH containing more small peptides and high antioxidant activity.

## Introduction

Proteins from plant sources have generated interest and are in high demand by consumers as they provide good alternatives with health benefits. Rice brans are waste products from the milling of rice, approximately 8–10% of total paddy rice weight^1^, which are generally used in low-value products as animal meal and fertilizer. Thailand is the world’s rice producer and exporter of jasmine rice or known as Khao Dok Mali 105 cultivar. The characteristic of jasmine rice, known its for long grain, tender texture and sweet-scented aroma. The major aroma compound of jasmine rice has been identified as 2-acetyl-1-pyrroline (2AP) that a perceived criteria for good quality rice^2^. The composition of rice bran provides about 14.7% protein, 20.9% fat, 52.3% carbohydrate and some other components^1^. The extraction of valuable protein components from rice bran could be increased to provide functional ingredients and flavor enhancers and also reduce disposal, thereby lowering environmental pollution.

Enzymatic hydrolysis is a mild process which has been used to prepare food protein hydrolysates, with fewer undesirable side effects. Enzymatic treatment raises the extraction yield and enhances the content of bioactive compounds. These bioactive hydrolysates can be food proteins which have potential as natural antioxidants^3^. Protein hydrolysates with antioxidant potential can inactivate scavenging free radicals acting as hydrogen donors or electron donors, reducing hydroperoxides and reactive oxygen species^4^. Alcalase is an endopeptidase which breaks peptide bonds from C-terminal amino acids and has the highest efficacy resulting in the highest antioxidant activity, whereas flavourzyme is an endo- and exopeptidase that breaks the N-terminal of peptide chains^5^. Certain mixtures of proteolytic enzymes such as alcalase and flavourzyme are appropriate for preparing rice bran protein hydrolysates due to their high productivity and high antioxidant activity^6^. However, a major barrier to the application of protein hydrolysates in functional foods and health products is the bitter taste from alcalase. Significant reductions in bitterness can be achieved using exo-proteinase digests such as with flavourzyme. Co-enzymatic hydrolysis applying a combination of flavourzyme and alcalase can be optimized using response surface methodology (RSM). RSM is a mathematical and statistical analysis technique capable of improving the performance of mixture of enzyme and hydrolysis time factors that influence responses, to optimize the protein hydrolysis conditions.

In order to rice bran protein hydrolysate to be applied as functional food ingredients, they must produce their high bioactive activities. Even if the improvement of peptide bioactivities by combinations of several enzymes may contribute to the increase of antioxidant activity, there is no data available to study the proportion suitability of mixtures enzymes together. Based on the above rationale, this study was carried out to determine the optimal conditions for obtaining rice bran protein hydrolysates by co-enzymatic hydrolysis of jasmine rice bran. The ratio of flavourzyme to alcalase (Fl : Al) and hydrolysis time were optimized using RSM. In addition, the goal was to validate the optimized conditions based on combination of the degree of hydrolysis, protein content, molecular weight distribution and antioxidant activities.

## Results and discussion

### Effect of Fl:Al ratio and hydrolysis time on the DH of JBH

**Figure 1:**
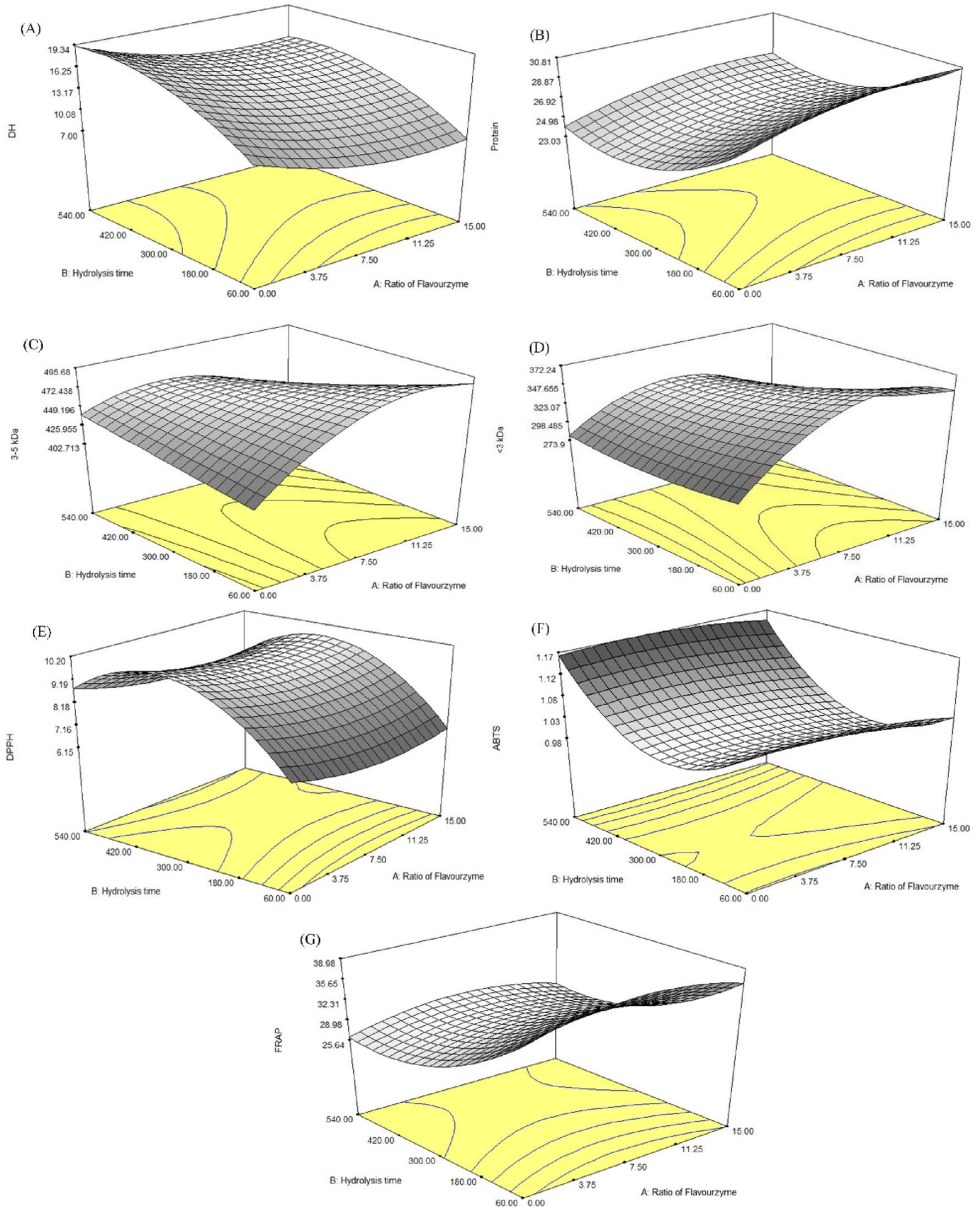
Response surface plots for effect of independent variables on DH (**A**), protein content (**B**), MMW peptides (3–5 kDa) (**C**), LMW peptides (< 3 kDa) (**D**), IC_50_ of DPPH (**E**), IC_50_ of ABTS (**F**) and FRAP (**G**).

The JBH samples had a DH in the range of 7.09–19.31% (Table 1). The model showed a highly significant value (p < 0.0001) (Table 3). The DH was strongly influenced by the linear term of the Fl:Al ratio (X_1_) and hydrolysis time (X_2_) (p < 0.0001) and the quadratic term of the Fl:Al ratio $({\mathrm{X}}_{1}^{2})$ and time ${(\mathrm{X}}_{2}^{2})$ (p < 0.001), whereas the interaction of the two factors (X_1_X_2_) was not significant (p ≥ 0.05). The statistical analysis showed a lack of fit of the model (p = 0.57), reflected in a relatively low determination coefficient (R^2^ = 0.96) (Table 3). The regression coefficient (β) values showed that the hydrolysis time (X_2_) had a greater influence on DH than the Fl:Al ratio. The response plot for DH with respect to the Fl:Al ratio and hydrolysis time is presented in Fig. 1A. The response plot shows that the Fl:Al ratio increased, the DH decreased.

**Table 1:** Experimental design of factorial design parameters obtained for independent variables: X_1_ (alcalase to flavourzyme ratio, w/w), X_2_ (time, min) and experimental values of responses: DH (%), protein content (%) and area of molecular weight (mAU*S).

Factors		DH (%)	Protein (%)	Area of molecular weight (mAu*S)		
Ratio of Fl:Al (%)	Hydolysis time (min)			5–10 kDa	3–5 kDa	< 3 kDa
0:100.0	60	12.05	39.51 ± 1.33	673.62 ± 7.07	414.92 ± 4.24	279.24 ± 7.28
7.5:92.5	60	7.09	42.36 ± 0.63	770.12 ± 12.03	469.32 ± 1.41	367.24 ± 3.54
15.0:85.0	60	7.79	42.17 ± 0.24	724.27 ± 21.92	505.51 ± 5.66	355.01 ± 8.49
0:100.0	300	17.74	34.40 ± 0.14	683.21 ± 2.83	419.65 ± 4.24	272.42 ± 1.41
7.5:92.5	300	12.90	37.22 ± 0.18	857.67 ± 23.33	482.48 ± 5.66	348.53 ± 6.36
15.0:85.0	300	12.79	38.76 ± 0.14	659.86 ± 16.26	416.59 ± 2.12	292.44 ± 13.44
0:100.0	540	19.31	36.51 ± 0.51	751.85 ± 8.49	438.20 ± 7.07	285.51 ± 1.41
7.5:92.5	540	14.50	38.09 ± 0.10	752.75 ± 2.83	454.44 ± 10.61	309.78 ± 7.07
15.0:85.0	540	14.77	38.19 ± 0.25	741.07 ± 7.07	413.75 ± 4.95	288.42 ± 7.78
7.5:92.5	300	13.80	36.57 ± 0.14	834.09 ± 4.61	470.83 ± 1.83	332.26 ± 12.57
7.5:92.5	300	14.60	37.87 ± 0.14	857.88 ± 2.81	488.65 ± 9.23	353.23 ± 5.77
7.5:92.5	300	14.40	38.76 ± 0.24	815.94 ± 26.64	451.62 ± 0.13	339.89 ± 16.28
7.5:92.5	300	14.20	37.84 ± 0.12	835.33 ± 15.58	486.88 ± 11.72	336.36 ± 1.35

Note: All values are Mean ± SD of three individual determinations.

This might be due to flavourzyme being less active when in combination with alcalase or hydrolyzing rice bran to a limited extent. This result is in agreement with Wang et al.^7^ who demonstrated a higher DH for the cleavage of protein from tree peony seeds by alcalase than by flavourzyme, due to alcalase having very broad specificity to break peptides. In addition, rice bran protein hydrolysate appears to be a more desirable substrate for alcalase given the higher DH observed using only alcalase. During the initial stage of the enzyme reaction (60 min), DH increased at a rapid rate and then decreased slightly with an increase in reaction time (Fig. 1A). This reduction of the rate of enzyme hydrolysis might be due to substrate limitation and the reduction of enzyme activity. The same has been found for hydrolysis of peptides from pecan meal using alcalase^8^.

### Effect of Fl:Al ratio and hydrolysis time on protein content of JBH

The protein content of JBH samples was determined by the combustion method and was in the range from 34.40 to 42.36 g/100 g sample, as presented in Table 1. The response plot for protein content shows that the Fl:Al ratio (X_1_) had a major influence on protein content: as the ratio increased, the amount of protein increased (Fig. 1B). Alcalase is an endopeptidase, which breaks peptide bonds from C-terminal amino acids, whereas Flavourzyme is an endo- and exopeptidase that breaks the N-terminal of peptide chains^5^. This indicates that Flavourzyme could also increase the number of N-terminal sites for the action of the exopeptidase. However, as hydrolysis time increased, the protein content decreased slightly (Fig. 1B). These result might be occurred by Maillard reaction, the increase of α-free amino acid via hydrolysis could interact with carbonyl group from lipid oxidation or sugar^9^. However, JBC may still have residue of 2.42 ± 0.16% fat content and carbohydrate content ^1^.

### Effect of Fl:Al ratio and hydrolysis time on MW distribution of JBH

The MW distribution of rice bran hydrolysates varied according to Fl:Al ratio and hydrolysis time, as presented in Table 1. The MW of JBH was categorized as high molecular weight (HMW, 5–10 kDa), medium molecular weight (MMW, 3–5 kDa) or low molecular weight (LMW, less than 3 kDa): 673.52–857.88, 413.75–505.51 and 272.42–367.24 mAU*s, respectively. The regression coefficients for MMW and LMW models of the multiple regression are presented in Table 3. As shown in Fig. 1C and D, as the Fl:Al ratio was increased, the proportion of MMW (3–5 kDa) and LMW (< 3 kDa) peptides increased. Exopeptidases can attack different active sites of a polypeptide, resulting in an increase in the content of MMW and LMW. The MW distribution of protein is related to its antioxidant activity^10,11^.

### Effect of Fl:Al ratio and hydrolysis time on antioxidant activity of JBH

The antioxidant activity of rice bran protein hydrolysates was determined by DPPH, ABTS, FRAP and H_2_O_2_ assays (Table 2). According to statistical analysis, the DPPH, ABTS and FRAP values were identified as significant model terms whereas for H_2_O_2_ was found to be non-significant (Table 3). The adj. R^2^ for DPPH, ABTS and FRAP was 0.63, 0.76 and 0.84, respectively, with a non-significant value for lack of fit (p ≥ 0.05) for all responses, showing a significant and good fit with the experimental data and having less variation.

**Table 2:** Antioxidant activity of rice bran protein hydrolysates with various independent factors.

Factors		Antioxidant activities			
Ratio of Fl:Al (%)	Hydolysis time (min)	IC_50_ of DPPH (mg/mL)	IC_50_ of ABTS (mg/mL)	FRAP (mmol FeSO_4_/100 g)	H_2_O_2_ (mM Trolox/g)
0:100.0	60	8.03 ± 0.56	1.10 ± 0.02	715.98 ± 19.67	262.11 ± 9.53
7.5:92.5	60	5.13 ± 0.34	1.04 ± 0.02	817.74 ± 49.43	258.42 ± 6.60
15.0:85.0	60	6.42 ± 0.55	1.06 ± 0.03	712.53 ± 18.92	241.27 ± 4.14
0:100.0	300	9.90 ± 0.39	1.03 ± 0.02	531.07 ± 15.28	192.87 ± 3.71
7.5:92.5	300	8.91 ± 0.75	1.05 ± 0.04	652.18 ± 13.11	240.60 ± 3.33
15.0:85.0	300	9.86 ± 0.51	0.99 ± 0.01	551.15 ± 19.86	220.10 ± 2.65
0:100.0	540	7.96 ± 0.14	1.15 ± 0.03	532.54 ± 8.62	222.12 ± 4.82
7.5:92.5	540	9.18 ± 0.52	1.20 ± 0.02	562.55 ± 20.52	205.98 ± 3.71
15.0:85.0	540	7.95 ± 0.36	1.13 ± 0.01	554.61 ± 33.31	220.10 ± 8.80
7.5:92.5	300	8.28 ± 0.24	0.97 ± 0.02	616.83 ± 30.54	211.36 ± 3.71
7.5:92.5	300	9.58 ± 0.44	1.01 ± 0.01	575.73 ± 20.03	197.24 ± 9.61
7.5:92.5	300	9.90 ± 0.09	1.07 ± 0.01	608.78 ± 10.48	206.32 ± 8.80
7.5:92.5	300	9.85 ± 0.38	1.03 ± 0.02	589.43 ± 11.34	202.96 ± 4.06

Note: All values are Mean ± SD of three individual determinations.

**Table 3:** Regression coefficient (β) and adjusted coefficient of determination (adj. R^2^) of the predicted second-order model for the response variables.

Responses	Regression coefficients (β)						
	DH	Protein	3–5 kDa	< 3 kDa	DPPH	ABTS	FRAP
**Constant**							
X_0_	9.61	41.43	405.57	287.67	5.44	1.12	793.24
**Linear**							
X_1_	− 0.83	0.48	15.96	18.15	− 0.04	1.78 × 10^–3^	18.05
X_2_	2.47	− 0.03	0.06	− 0.08	0.02	− 8.52 × 10^–4^	− 1.30
**Quadratic**							
${\mathrm{X}}_{1}^{2}$	0.03	**− **0.02	**− **0.65	**− **0.86	–	–	**− **1.15
${\mathrm{X}}_{2}^{2}$	**− **0.16	4.41 × 10^–5^	–	1.32 × 10^–4^	**− **3.51 × 10^–5^	1.74 × 10^–4^	1.47 × 10^–3^
**Interaction**							
X_12_	–	–	− 0.02	− 0.01	–	–	–
Adj. R^2^	0.9629	0.8742	0.6870	0.8642	0.6311	0.7585	0.8981
F-value	78.76	21.84	7.58	16.28	7.84	13.56	27.45
P-value	< 0.0001	0.0002	0.0079	0.0010	0.0070	0.0011	0.0001
Lack of fit	0.5696	0.5142	0.2716	0.1394	0.2368	0.7565	0.5998

### DPPH radical scavenging activity

The IC_50_ value is a parameter widely used to measure the antioxidant activity. It is calculated as the concentration of antioxidants needed to decrease the initial DPPH concentration by 50%^12,13^. The half-maximal inhibitory concentration (IC_50_) of DPPH radical scavenging activity in JBH samples showed variation, ranging from 5.13 to 9.90 mg/mL. The DPPH radical scavenging ability indicates that the JBH possessed the capacity to donate hydrogen atoms and electron^14^. The result shown in Fig. 1E demonstrates that the IC_50_ of DPPH scavenging activity was not affected by the Fl:Al ratio. The response plot shows that as hydrolysis time (X_2_) increased, the IC_50_ of DPPH increased up to 300 min which means the result of the sample was low activity. Then, the lower IC_50_ value indicated the higher antioxidant activity. This might be due to initial breakdown of peptide bonds in the hydrolysate, resulting in more HMW peptides than LMW peptides. This result was correlated with the regression model for an increase in < 3 kDa peptides as hydrolysis time increased. This might be attributed to the fact that peptides with LMW could more easily react with free radicals^4^. The potency of the hydrolysate’s DPPH radical scavenging activity depends on the size of peptides and type of enzyme^14^.

### ABTS radical scavenging activity

The ABTS radical scavenging assay, based on electron transfer and hydrogen atom transfer, can be performed to assess the radical scavenging activity of both the hydrophilic and hydrophobic compounds of protein hydrolysates^3,15^. As shown in Fig. 1F, as hydrolysis time was increased, the IC_50_ of ABTS decreased and then increased with a further increase in hydrolysis time. This might be due to the donation of electrons and hydrogen atoms of peptides in the hydrolysate liberated from protein during hydrolysis, resulting in an increase in the ability to scavenge ABTS. However, after excessive hydrolysis time, peptides were broken down into free amino acids, thus decreasing ABTS activity^16^. The ABTS radical scavenging ability can be explained by the DH, amino acid composition of peptide chains and the MW of peptides^5,17^.

### FRAP

The FRAP assay is used to determine a substance’s ability to donate electrons to convert ferric ions (Fe^3+^) to the ferrous form (Fe^2+^). FRAP varied for the different JBH treatments between 532.54 and 817.74 mmol FeSO_4_/100 g sample. Increasing the hydrolysis time significantly decreased FRAP (Fig. 1G). For a hydrolysis time of 60 min, more HMW peptides than LMW peptides or free amino acids were found, which could be responsible for hydrolysate being a poorer source of reducing electrons and protons^4^. After excessive hydrolysis time, FRAP increased slightly. These results are inconsistent with the study of Olagunju et al.^14^ who stated that HMW peptides (> 10 kDa) exhibit better FRAP than LMW peptides (< 5 kDa). In addition, Phongthai et al.^4^ reported that the presence of Tyr and Trp in a peptide indicates greater FRAP to reduce or donate electrons.

### H_2_O_2_ scavenging activity

The H_2_O_2_ scavenging activity of JBH samples prepared using different Fl:Al ratios and hydrolysis times ranged from 192.87 to 262.11 mM Trolox/g sample (Table 1). The JBH samples hydrolyzed for 60 min had the highest H_2_O_2_ scavenging activity, compared with those hydrolyzed for longer at the same Fl:Al ratio. It was noticed that as hydrolysis time increased, more short peptide chains or free amino acids were obtained, resulting in low H_2_O_2_ scavenging activity^18^.

Interestingly, the Fl:Al ratio had effect on the antioxidant activities, indicating that these enzymes are more effective for obtaining both hydrophobic amino acid (e.g. Ala, Val, Leu, Iso, Pro, Phe, Try, Cys and Met) and hydrophilic amino acid (e.g. Ser, Thr, Asn, Glu, His and Tyr) sequences in protein hydrolysate samples^6^. These results indicate that the combination of flavourzyme and alcalase can enhance antioxidant activity, by producing peptides with different specific amino acids. Tang et al.^6^ demonstrated that *Tenebrio molitor* larvae hydrolysate obtained using a mixture of alcalase and flavourzyme exhibits the highest antioxidant activity. According to Ambigaipalan et al.^5^, date seed hydrolysates obtained using an alcalase and flavourzyme combination exhibited the highest antioxidant activity. However, Kumar and Roy^19^ reported that hydrolysates obtained using an enzyme combination showed lower values for DPPH activity and FRAP than those obtained using alcalase and flavourzyme alone.

### Optimization and verification of protein rice bran hydrolysate

**Figure 2:**
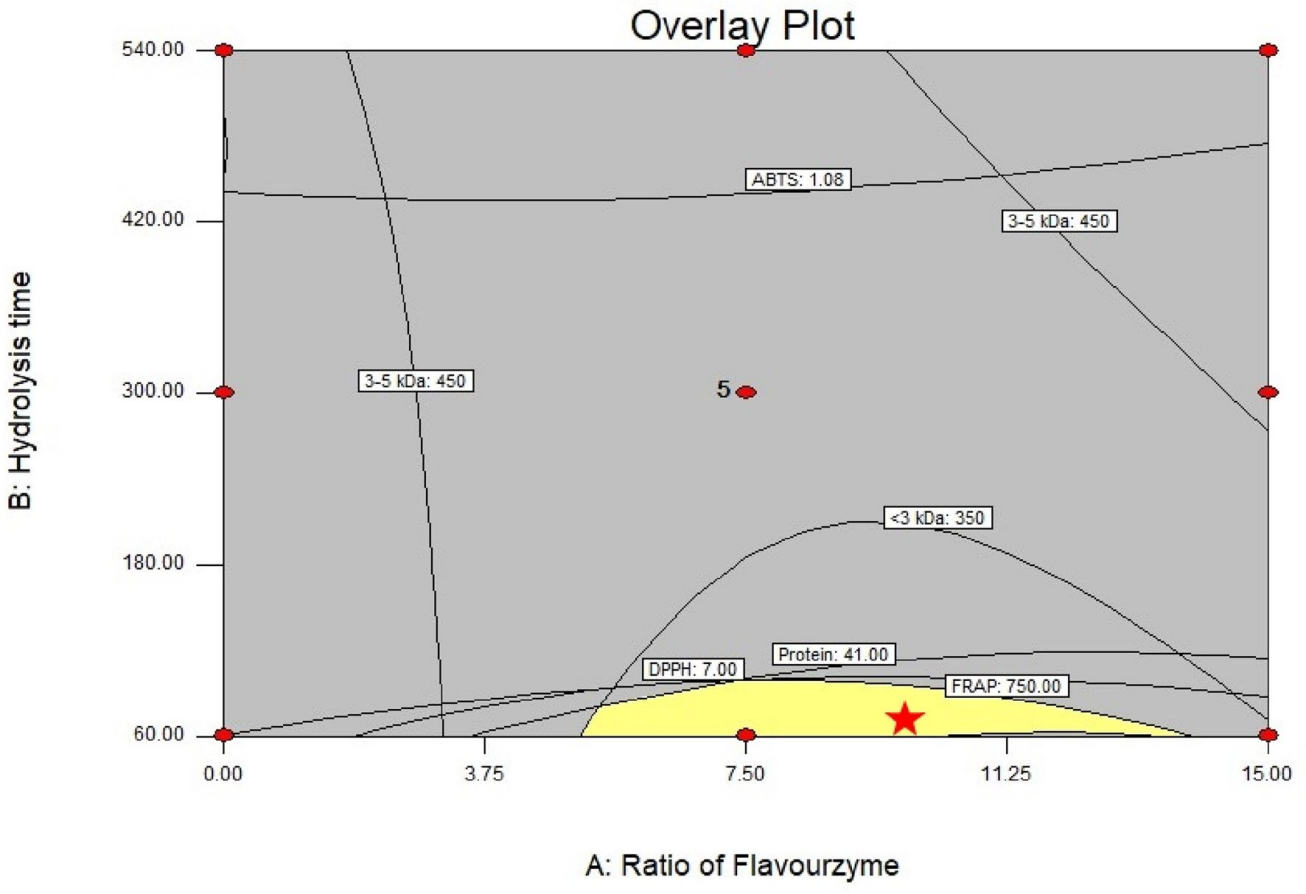
Overlay plot of response surface demonstrating the optimum formula for jasmine rice bran protein hydrolysates with flavourzyme : alcalase ratio (% w/w) and hydrolysis time (min).

According to the RSM analysis, rice bran protein hydrolysate that is considered to be desirable should at least provide these properties: (1) a high protein content, (2) high antioxidant activity and (3) a high content of LMW protein. The optimal conditions were an Fl:Al ratio of 9.81 : 90.19 and hydrolysis time of 60 min; the desirability value for these conditions was 80.6% which was located in the optimal area as shown in Fig. 2.

Under the optimum conditions for producing the protein hydrolysate, obtained values were in agreement with the predicted values; the difference error ranged from 0.42 to 8.42% (Table 4). The corresponding response values obtained from the actual data and those predicted from the models were similar.

**Table 4:** Experimental data of the validation at optimal extraction conditions.

Dependent variables	Predicted value	Experimental value	% Difference
DH (%)	7.15	7.18 ± 0.12	−0.42
Protein (%)	42.50	41.73 ± 1.08	− 1.80
IC_50_ of DPPH (mg/mL)	6.15	6.59 ± 0.20	− 8.43
IC_50_ of ABTS (mg/mL)	1.07	0.99 ± 0.01	7.48
FRAP (mmol FeSO_4_/100 g)	787.39	724.81 ± 12.99	7.95
MW 3–5 kDa (mAU*s)	442.57	449.05 ± 9.45	1.46
MW < 3 kDa (mAU*s)	319.71	322.35 ± 21.15	0.82

### Amino acid composition of optimum rice bran protein hydrolysate

The amino acid composition for the optimum JBH conditions are presented in Table 5. It was observed that the JBH sample had a high content of Glu, Arg and Asp: 338.90, 99.10 and 91.22 mg/g protein, respectively. These results are consistent with those of Xiao et al.^20^ who found that Glu, Asp and Arg were the most abundant amino acids in rice bran protein hydrolysate. However, the protein hydrolysate of some plants such as mung bean is rich in Asp, Glu and Pro ^21^. The high content of Glu and Asp could have been due to their abundance in the plant protein. The total amount of essential amino acids in the JBH sample was 262.50 mg/g protein; the recommendation for human nutrition is approximately 277 mg/g protein, suggesting that JBH could enhance or provide suitable protein nutrition^22^. The sample showed a high content of flavor enhancers, especially Glu (338.90 mg/g), followed by Asp (91.22 mg/g), Ala (59.85 mg/g) and Gly (37.34 mg/g). The JBH had a hydrophobic amino acid content of 266.98 mg/g protein. Samaranayaka and Li-Chan^23^ obtained a protein hydrolysate containing Ala, Lys, Pro, Leu, His, Tyr and Met, suggesting that hydrophobic amino acids could contribute to high antioxidant activity. In addition, the JBH contained 52.86 mg/g protein of aromatic amino acids, which have been reported to improve the radical scavenging activity of peptides by donating electrons to electron-deficient radicals^7^.

**Table 5:** Total amino acid compositions of jasmine rice bran protein hydrolysate obtained at optimum condition.

Amino acid	Content (mg/g protein)
Aspartic acid (Asp)	91.22 ± 0.21
Threonine (Thr)	22.58 ± 0.37
Serine (Ser)	35.97 ± 0.61
Glutamic acid (Glu)	338.90 ± 8.54
Proline (Pro)	33.01 ± 0.07
Glycine (Gly)	37.34 ± 3.82
Alanine (Ala)	59.85 ± 4.51
Cysteine (Cys)	1.76 ± 0.44
Valine (Val)	37.52 ± 2.49
Methionine (Met)	4.74 ± 0.25
Isoleucine(Ile)	22.72 ± 1.57
Leucine (Leu)	54.51 ± 4.80
Tyrosine (Tyr)	25.68 ± 3.11
Phenylalanine (Phe)	27.19 ± 0.19
Histidine (His)	19.45 ± 1.92
Lysine (Lys)	46.35 ± 1.16
Arginine (Arg)	99.10 ± 5.65
Essential amino acids (EAAs)^a^	262.50
Aromatic amino acids (AAAs)^b^	52.86
Hydrophobic amino acids (HAAs)^c^	266.98

^a^EAAs are Thr, Cys, Val, Met, Ile, Leu, Tyr, Phe, His and Lys.

^b^AAAs are Phe and Tyr.

^c^HAAs are Ala, Val, Ile, Leu, Tyr, Phe, Pro, Met and Cys.

## Conclusion

The addition of flavourzyme improved the efficiency of producing jasmine rice bran protein hydrolysate. An Fl:Al ratio of 9.81 : 90.19, at an enzyme concentration of 2.84% (w/w) and 60 min hydrolysis time were chosen as the optimum conditions for obtaining JBH. These conditions provided a high protein content, high antioxidant activity and a high content of LMW and MMW protein. Therefore, JBH could serve as a potential source of functional food ingredients for health promotion.

## Materials and methods

### Materials

Jasmine paddy rice cultivar seeds (Khao Dok Mali 105, *Oryza sativa* L.) were kindly provided by the Lanna Rice Research Center, Chiang Mai University, Thailand (http://lanna-rice.cmu.ac.th/). The identification was done according to the Rice Department, Ministry of Agriculture and Cooperatives, Thailand. The use of plants in the present study complies with international, national and/or institutional guidelines. The paddy rice was de-husked and milled to produce jasmine rice bran (JB) with a laboratory husker. The JB was stored in aluminum bags and kept at − 18 °C until use.

Alcalase ≥ 2.4 U/g (declared enzyme activity 2.4 AU/g) and flavourzyme ≥ 500 U/g (declared enzyme activity 500 LAPU/g) were obtained from Sigma-Aldrich (St. Louis, MO, USA). AU is defined as Anson units; LAPU is defined as leucine aminopeptidase units^24^. The gel filtration calibration kit (low molecular weight) was purchased from Cytiva (Marlborough, MA, USA). All the other chemicals and reagents used were purchased as the analytical grade.

### Preparation of jasmine rice bran protein concentrate (JBC)

The JB was defatted as described by Hunsakul, Laokuldilok, Prinyawiwatkul and Utama-ang^25^. In brief, the JB and 95% ethanol with the ratio of 1:3 (w/v) were continuously stirred for 30 min, centrifuged at 4800×*g* for 10 min. The precipitate was collected and re-extracted twice and then dried at 45 ± 2 °C until the moisture content remained constant. Then, 100 g of defatted rice bran was dispersed in 1 L of 0.05 M NaOH. After continuous stirring for 1 h, the dispersion was centrifuged at 2100×*g* at 4 °C for 15 min (Sorvall Super T21; GMI, Ramsey, MN, USA) and solubilized bran was collected. The pH of the solubilized bran was adjusted to 4.0 with 1 M HCl and rested at 4 °C for 1 h and then centrifuged at 2100×*g*. The pH of the precipitate was adjusted to 7.0 with 1 M NaOH. The suspension of JBC was lyophilized and stored at − 18 °C until use. The protein content of JBC was 62.27 ± 1.63%.

### Production of jasmine rice bran protein hydrolysate (JBH) using combined enzymes

The activities of both enzymes were generally determined before used, in which the flavourzyme activity was 500 LAPU/g and the alcalase activity was 2.4 AU/g. The experimental design was carried out following a 3-level factorial design with five replicates at the center point, consisting of 13 treatments including four center point replications (Table 1).

In brief, The JBC sample of 4 g of protein was mixed with 100 mL of distilled water and heated at 60 °C for 10 min. The pH of the suspension was adjusted to 7.5 using 1 N Na_2_HPO_4_ then the experimental design was followed. The reaction was started by adding 2.84% protein (enzyme/protein substrate, w/w) with flavourzyme and alcalase at various ratios from 0 : 100 (6.82 AU/ 100 g of alcalase) to 15 : 85 (213 LAPU/ 100 g of flavourzyme and 5.79 AU/100 g of alcalase) (X_1_) and hydrolysis time was allowed to proceed for 60–540 min (X_2_).The hydrolysis reaction was terminated by placing the reaction mixture into a water bath at 95 °C for 10 min. The supernatant of protein was centrifuged at 4000×*g* for 15 min at 4 °C, and the pH adjusted to pH 7 before freeze drying. The protein suspension was lyophilized and stored at − 18 °C until use.

### Degree of hydrolysis (DH)

The DH was investigated according to the methodology of Adler-Nissen^26^. DH values are determined as the percentage of peptide bonds broken in relation to the total number of peptide bonds in the substrate studied (h_tot_). The DH values were calculated using the following equation.

$$\mathrm{DH }\left(\mathrm{\%}\right)=B\times {N}_{b}\times \frac{1}{\propto }\times \frac{1}{MP}\times \frac{1}{{h}_{tot}}\times 100$$

where B = the base consumption (mL) used to control the pH during the reaction, N_b_ is the normality of the base (1 N NaOH), α is the average degree of dissociation of the α-NH_2_ groups during hydrolysis (1/α = 1.13 for alcalase and flavourzyme), MP = the mass of protein (N × 5.95) in g, and h_tot_ is defined as 8.4 meq/g rice bran protein.

### Protein content

The protein content in JBH samples was determined by the combustion technique (Nitrogen combustion-FP-528, Leco, Germany). The percentage of protein was calculated using a conversion factor of 5.95.

### Antioxidant activity

#### DPPH radical scavenging activity

DPPH radical scavenging activity was determined according to the method of Phongthai et al.^4^. First, 150 µL of hydrolysate sample was dissolved in distilled water and then mixed with 150 µL of DPPH solution (0.1 mM DPPH in 95% ethanol). The mixture was incubated at ambient temperature for 30 min. The absorbance of samples was read at 517 nm. The result for each sample was expressed as an effective concentration that causes 50% scavenging of the initial radical concentration (IC_50_, mg/mL).

#### ABTS radical scavenging activity

ABTS radical scavenging activity was determined according to Re et al.^27^ with slight modifications. The 7 mM ABTS radical solution was mixed with 2.45 mM K_2_S_2_O_8_ at a ratio of 1 : 1 and then kept in the dark at room temperature for 12–16 h before use. The absorbance of the mixed solution was adjusted to 0.7 ± 0.02 using distilled water prior to testing. ABTS free radical scavenging activity was reported as the IC_50_ of the sample (mg/mL).

#### Ferric reducing activity power (FRAP)

FRAP was assessed according to the method reported by Benzie and Strain^28^ with some modification. A fresh FRAP solution was prepared by combining 10 mM TPTZ (dissolved in 40 mM HCl), 20 mM FeCl_3_ and 0.3 M acetate buffer at a ratio of 1 : 1 : 10 (v : v : v) and incubated at 37 °C until use. The JBH samples (20 µL) were dissolved in distilled water at a concentration of 4 mg/mL, mixed with the FRAP reagent (180 µL) and then incubated at 37 °C for 15 min. FeSO_4_ solutions (0–700 µM) were used to obtain standard curves. The absorbance was read at 593 nm and the FRAP expressed in mmol FeSO_4_/100 g sample.

#### Hydrogen peroxide (H_2_O_2_) scavenging activity

The H_2_O_2_ activity was determined according to the method of Chandrasekara and Shahidi^29^ with slight modification. The JBH sample 1 mg was dissolved in 1 mL of 0.1 M phosphate buffer, pH 7.4 (at a concentration of 1 mg/mL). The sample (0.6 mL) was mixed with 900 µL of 40 mM hydrogen peroxide (dissolved in 45 mM phosphate buffer at pH 7.4) and allowed to react for 1 min at 25 °C. The absorbance was recorded at 230 nm. A blank was prepared in the same manner but with buffer instead of sample. The hydrogen peroxide scavenging activity was calculated and reported as mg Trolox/g sample.

#### Molecular weight (MW) distribution

The MW distribution of the JBH samples was determined using the method of Delgado et al.^30^ with slight modifications. The HPLC system was comprised of an SRT-C SEC-300 size exclusion column (5 μm, 4.6 × 300 mm, Sepax Technologies, USA) and a Shimadzu photodiode array detector. Ten microliters of sample solution (3 mg/mL dissolved in 0.05 M phosphate buffer (pH 7.0) in 0.15 M sodium chloride) was injected, the flow rate set at 0.25 mL/min and the reaction monitored at 280 nm. MW calibration curves were provided by N-hippuryl-His-Leu (0.43 kDa) followed by aprotinin (6.50 kDa), ribonuclease (13.70 kDa), carbonic anhydrase (29.00 kDa), ovalbumin (44.00 kDa) and conalbumin (75.00 kDa).

#### Amino acid composition

In brief, 50 mg of each sample was dissolved in 6 N HCl with 1% phenol at 110 °C for 24 h. After being hydrolyzed, the samples were delivertized with phenyl isothiocyanate for 10 min at 25 °C. The prepared samples were injected into a Shim-pack Amino-Na column (46 mm × 100 mm, 5 µm) with an RF-20A detector (Shimadzu, Japan) and monitored at 280 nm. The flow rate was set at 0.4 mL/min. The calibration curves for determining amino acid composition were constructed using an amino acid standard (Sigma-Aldrich, USA).

### Experimental design and statistical analysis

The independent variables studied were the Fl:Al ratio (X_1_), ranging from 0 : 100 to 15 : 85 w/w at an enzyme concentration of 2.84%, and hydrolysis time (X_2_), which ranged from 60 to 540 min. Regression coefficients of variables were analyzed by a quadratic regression polynomial following the equation:

$$Y={\beta }_{0}+\sum_{i=1}^{2}{\beta }_{i}{x}_{i}+\sum_{i=1}^{2}{\beta }_{ii}{x}_{i}^{2}+\sum_{i=1}^{2}\sum_{j=2}^{2}{\beta }_{ij}{x}_{i}{x}_{j}$$

where Y is the response variable; β_0_ is a constant; β_i_, β_ii_ and β_ij_ are the linear, quadratic and interaction coefficients, respectively; and x_i_ and x_j_ are the independent variables.

All data were used to perform the RSM to optimize hydrolysis using Design-Expert software (version 6.0.2; Stat-Ease Inc., USA). The statistical analyses assessed the analysis of variance (ANOVA), adjusted regression coefficients (adj. R^2^) and lack of fit.
